# Applications and Challenges of Supercritical Foaming Technology

**DOI:** 10.3390/polym15020402

**Published:** 2023-01-12

**Authors:** Yujin Zhou, Yingrui Tian, Xiaowei Peng

**Affiliations:** 1 College of Physical Education, Wuhan Sports University, Wuhan 430079, China; 2 College of Science, Wuhan University of Science and Technology, Wuhan 430081, China; 3 School of Materials and Metallurgy, Wuhan University of Science and Technology, Wuhan 430081, China

**Keywords:** supercritical foaming technology, foaming agent, application, development, challenge

## Abstract

With economic development, environmental problems are becoming more and more prominent, and achieving green chemistry is an urgent task nowadays, which creates an opportunity for the development of supercritical foaming technology. The foaming agents used in supercritical foaming technology are usually supercritical carbon dioxide (ScCO_2_) and supercritical nitrogen (ScN_2_), both of which are used without environmental burden. This technology can reduce the environmental impact of polymer foam production. Although supercritical foaming technology is already in production in some fields, it has not been applied on a large scale. Here, we present a detailed analysis of the types of foaming agents currently used in supercritical foaming technology and their applications in various fields, summarizing the technological improvements that have been made to the technology. However, we have found that today’s supercritical technologies still need to address some additional challenges to achieve large-scale production.

## 1. Introduction

Foams are multiphase materials, one of which is in the gaseous state. They are versatile materials encountered in nature from bones to tree trunks, as well as everywhere in our daily life from our cars to beds [1]. Among the foam materials, polyurethane accounts for the largest share in terms of application scope and size. According to industry research, the polyurethane foam market is worth $42.9 billion in 2021 (Source: BlueWeave Consulting). As the market continues to expand, the requirements for the properties of foams continue to increase. Therefore, the choice of foaming technology is crucial. This has created an opportunity for the application of supercritical foaming technology. As the name implies, the foaming agents used in supercritical foaming technology are supercritical fluids (SCFs). SCFs are substances with pressure and temperature above the critical value, such as ScCO_2_ and ScN_2_. Processes involving SCFs are sustainable, environmentally friendly, and cost-effective [2]. In addition to foaming, SCFs can also be used for extraction, impregnation, particle coating, etc. Among these areas, foaming and extraction are the most studied. In the field of extraction, supercritical fluids play the most critical role. For example, seed oil is extracted by ScCO_2_ extraction [3,4]. In the field of foaming, SCFs are physical blowing agents (PBAs), which are greener than chemical blowing agents (CBAs) in the process of use. Therefore, supercritical foaming technology can reduce environmental pollution in the foaming process, which is in line with the concept of sustainable development. This technology is a promising approach to obtaining polymeric foams, which are widely used in sneaker midsole technology research. However, supercritical foaming technology is not widely used in industrial practice in areas other than sneakers.

**Figure 1:**
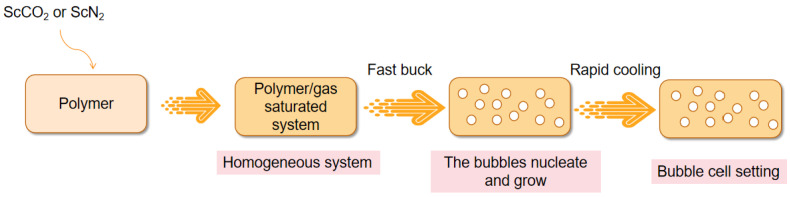
Flow chart of supercritical foaming technology.

Suh and colleagues first investigated polymer foaming using gases, such as CO_2_ and N_2_, in the supercritical state in the 1980s, which marked an important advance in foaming technology [5]. Supercritical foaming technology is a physical foaming and molding technology as well as a microporous foaming and molding technology. The flow chart of this technology is shown in Figure 1. In supercritical foaming, SCFs are used alone or in combination with other gases as foaming agents. The most commonly used foaming processes are intermittent foaming, extrusion, and injection molding. Among them, intermittent foaming is mainly used on a laboratory scale to provide good control over the processing variables [6]. Supercritical foaming technology is an effective method for preparing microporous plastics, which has been developed rapidly in the past 20 years. Compared with other chemical foaming technologies, the microporous plastic foam obtained by supercritical foaming technology has a smaller pore size, higher pore density, and better overall performance. Mitigating the greenhouse effect has long been a challenge for mankind. Carbon dioxide is the most important greenhouse gas, and at present, the amount of carbon dioxide in the atmosphere is increasing year by year. Atmospheric CO_2_ concentrations measured in May of this year reached 421 ppm (Source: National Oceanic and Atmospheric Administration and Scripps Institution of Oceanography scientists). Carbon dioxide can be utilized as a foaming agent for supercritical foaming technology, which contributes to mitigating the greenhouse effect to a certain extent. Therefore, supercritical foaming technology is a promising area for research and development. Recent trends include the preparation of microporous scaffolds [7,8,9] and the production of rubber foams [10]. However, there are many shortcomings in this technology. The current supercritical foaming technology is costly, the finished product size control is still lacking, and the market demand has not yet been released. If supercritical foaming technology is used on a large scale in the future, manufacturers will be able to obtain foam products with sizes, shapes, and densities that are more in line with actual needs. In addition, it will reduce the environmental impact of the production process.

Conventional chemical foaming technology is the use of chemical methods to produce gas to make plastic foaming: adding a chemical foaming agent in plastic, by heating it to produce gas and foaming; or using each plastic component of the chemical reaction between each other to release gas and foaming. This is not the case with supercritical foaming technology, which consists of two basic steps: (a) absorption or dissolution of the gas in the polymer matrix under pressure to form a polymer gas solution, and (b) nucleation and growth of the bubbles at reduced pressure (which is the common path) or increased temperature (if adsorption is performed at relatively low temperatures) [1]. The difference between chemical foaming technology and supercritical foaming technology is mainly in the foaming agent used and the source from which the foaming agent gas is obtained. CBAs are organic or inorganic compounds that thermally decompose into gases that do not react with the polymer matrix, such as azodicarbonamide (ADA). PBAs are compounds that expand rapidly due to phase changes, such as SCFs [11]. Chemical foaming agents are obtained through chemical reactions, while physical foaming agents used in supercritical foaming technology are obtained by changing the state of the gas. Therefore, in terms of the foaming process alone, supercritical foaming technology is greener and more in line with the needs of people’s lives nowadays. For example, in the field of pharmacology, residual CBAs in drugs stimulate human cells, but the PBAs used in supercritical foaming technology avoid this shortcoming. However, in the food field, chemical foaming technology is often used in food packaging, because the foaming output of chemical foaming technology is larger and the cost is low.

The most important feature of supercritical foaming technology is that the foaming agent used is often ScCO_2_ and ScN_2_, which are non-toxic and harmless gases. On the one hand, it can reduce pollution to the environment, and on the other, it can also reduce the harm of the product to the human body. Chemical foaming agents decompose some harmful gases, such as ammonia (NH_3_), formamide (COHNH_2_), and other CN compounds at decomposition temperatures. COHNH_2_ is a toxic substance, if the product remains more than 10 ppm of COHNH_2_, the product can cause serious harm to the human body. The ScCO_2_ and ScN_2_ used in supercritical foaming technology do not cause harm to the human body [12]. In addition, the principle of this technology is simple, and it is a very promising foaming technology. However, the supercritical foaming technology lacks good development, the technology is not simple enough, and the control of bubble pores is lacking. This is because supercritical foaming, although conceptually simple, is a complex and dynamic process that requires a thorough understanding of the fundamentals of polymer science, thermodynamics, physics, and chemistry of solutions and interfaces, interacting species, and polymer materials and process engineering [1]. Therefore, the supercritical fluid foaming process needs to be strictly controlled to produce foam that meets expectations.

However, according to the present research, the control of bubble hole size has been achieved and applied to some extent, e.g., by increasing the affinity between the organic solvent and ScCO_2_ to obtain low density and small pore size foam [13]; supercritical foaming technology was coupled with a leaching process to overcome problems of pore size tuning of the supercritical foaming technique [14]. This shows that this foaming technique is still feasible, although the finished size is difficult to control. Simplified solutions for supercritical foaming technology are now also proposed by research teams, such as the developed resilient and innovative cellular foam injection molding (RIC-FIM) technology [15]. These present the basic conditions for the widespread use of supercritical foaming technology in the future.

Green chemistry is currently an important concern in production and achieving green chemistry is an urgent task at the moment. In addition, people are becoming more and more concerned about green and healthy lifestyles and have higher requirements for products. All these factors give great room for the development of supercritical fluid foam molding technology. In terms of ScCO_2_ foaming technology, it is non-toxic, green, and it also avoids damage to the earth’s ozone layer [12]. If the market demand for supercritical fluid foam molding technology is fully released in the future, people will have a greener life. However, there is no complete set of recycling mechanisms to deal with waste foam products. Further, if the products obtained through supercritical technology cannot be effectively recycled, then they cannot really be environmentally friendly. Secondly, the foaming agents used in supercritical foaming technology are limited to ScCO_2_ and ScN_2_. Moreover, it is an open question whether other supercritical fluids can be used for foaming. Our study focuses on supercritical foaming technology. First, we analyze the foaming agents used in supercritical foaming technology. Then, the current applications of this technology in various fields are analyzed in detail. Finally, we discuss the current favorable technological developments and future remaining research in supercritical foaming technology.

## 2. Types of Foaming Agents

With the development of the economy, environmental problems are becoming more and more prominent. Green chemistry is one of the important means to solve the current environmental problems. At the same time, people are paying more and more attention to a green and healthy life. Based on this, physical foaming agents are gradually replacing chemical foaming agents, mainly supercritical fluids [11]. Therefore, there is great room for the development of supercritical foaming technology. The choice of foaming agent is an important task of the supercritical fluid foaming process. The use of different foaming agents for foaming, the product obtained for each performance, and the direction of application will also be different. Supercritical fluid foaming agents should have the characteristics of high foaming efficiency, be non-toxic, be environmentally friendly, and have high thermal stability. Therefore, many countries will legislate to restrict the use of toxic supercritical fluids such as HFCs. SFCs, such as ScCO_2_ and ScN_2_, have become the best choice in PBA [11].

**Figure 2:**
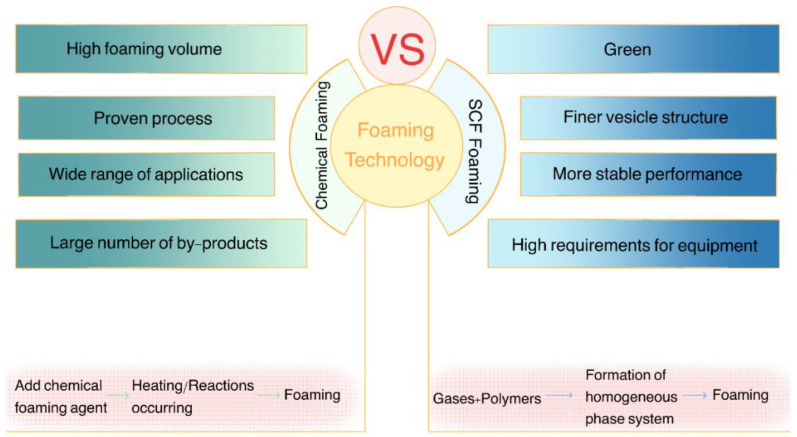
Comparison of supercritical foaming technology and chemical foaming technology.

**Figure 3:**
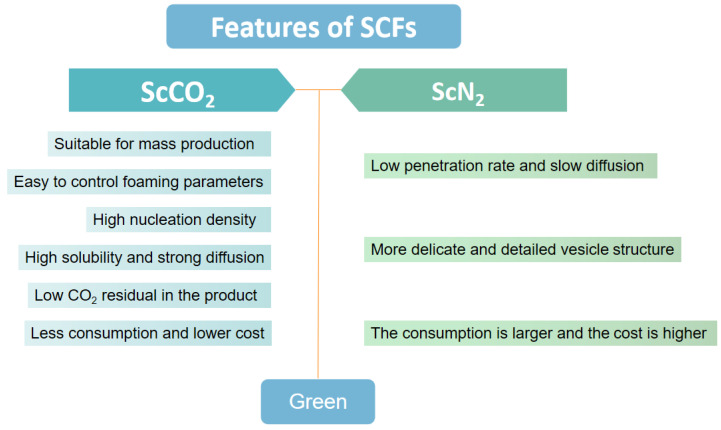
Characteristics of two supercritical foaming agents.

The characteristics and applications of foaming products when ScCO_2_ and ScN_2_ are used as foaming agents are listed here. The characteristics and main processes of chemical foaming technology and supercritical foaming technology are shown in Figure 2. The foaming characteristics of ScCO_2_ and ScN_2_ are shown in Figure 3.

### 2.1. ScCO_2_

ScCO_2_ is currently the most widely used foaming agent for supercritical foaming technology. It represents the main compressed fluid used today for the preparation of polymer foams with nano- or micron-sized pores in intermittent and continuous processes [13].

ScCO_2_ foaming technology has the following advantages. (a) This technology is suitable for mass production. (b) The foaming process does not release any toxic gas, which avoids damage to the earth’s ozone layer. (c) It is easy to control the foaming parameters or the addition of fillers can control the pore structure, ranging from hundreds of microns to a few microns in size. (d) ScCO_2_ has high solubility, strong diffusion ability, and high nucleation density in polymers, which facilitates the preparation of porous polymer materials. (e) ScCO_2_-assisted foaming offers significant advantages in terms of solvent-free processability of the material and loading of bioactive compounds in high yields during integration. (f) Low residual CO_2_ in the polymer matrix after foaming [12,14,16,17].

Supercritical foaming technology with ScCO_2_ as the foaming agent has a wide range of applications. First, it can assist the extrusion process for food production, pharmaceuticals, polymer foaming, the production of composite materials, etc. However, such a coupled technology is a new one and needs further research [18]. Second, it combines the advantages of processing materials from organic solvent solutions and ScCO_2_ intermittent foaming to prepare foams with uniform and controllable porosity, while also reducing solvent residues [13]. Third, the flexible porous silicone rubber-nanofiber nanocomposites produced by ScCO_2_ foaming can be used to harvest mechanical energy. For example, ScCO_2_ extrusion foaming is used to prepare strip-shaped samples to harvest energy from tires, and the ScCO_2_ foam injection molding method is used to obtain shoe-sole-shaped samples to harvest energy generated by human motion [16]. Fourthly, ScCO_2_ foaming is combined with the removal of solid porogen to obtain drug-loaded scaffolds to overcome the pore size adjustment problem of supercritical foaming technology [14].

### 2.2. ScN_2_

ScN_2_ is suitable for the production of high-density foam, such as foam injection molding using MuCell-type processes, polyethylene cable sheath extrusion, etc. The main features of supercritical fluid technology with N_2_ as a foaming agent are as follows. (a) The foam obtained is very fine. (b) Environmentally friendly, no toxic gas generation. (c) The N_2_ diffusion is slower and the formed foam is less likely to collapse. In addition, ScN_2_ is beneficial to the foaming of elastic materials. For example, Michaeli et al. found that the bubble pore structure of the EPDM rubber profile extrudate was more uniform when ScN_2_ was used as the foaming agent than when ScCO_2_ was used. The bubble pore size in the foam was smaller, the bubble pore density was higher, and the bubble pores were more uniform when ScN_2_ was used as a foaming agent compared to ScCO_2_. However, compared with ScN_2_, ScCO_2_ has higher solubility and a higher diffusion rate in the polymer [5]. At present, for supercritical foaming technology, the most widely used is ScCO_2_ foaming technology. ScN_2_ foam is mainly used in the sports field, such as sneaker midsole technology. Related technologies include Anta’s “Nitrogen Technology”, Li Ning’s “Boom (䨻)”, etc. These technologies all use supercritical nitrogen foaming technology. The main reason for this is that the foam product of N_2_ as a foaming agent is more delicate and uniform, making the product more functional. However, ScN_2_ is not used much in other applications. In supercritical foaming technology, the success rate of N_2_ for foaming is lower than that of CO_2_, and therefore the consumption of N_2_ is relatively greater. Therefore, in practice, the cost of N_2_ is higher than that of CO_2_ in terms of the foaming agent alone.

### 2.3. Co-Foamer

The foaming agents used in current supercritical foaming technology are generally only ScCO_2_ and ScN_2_, and basically no other supercritical fluids are used. However, the use of co-foaming agents is now introduced in the technical process to improve the foaming capacity of plastic materials. The use of co-foamer foaming means that other reagents are mixed with ScCO_2_ or ScN_2_ for foaming when supercritical fluid foaming is performed. Ethanol, water, and acetone can be used as co-foaming agents. For example, Tsivintzelis et al. used a CO_2_-ethanol mixture as a foaming agent for polycaprolactone (PCL) foaming and the results showed that the foam had a large expansion rate. Zhao et al. investigated PVOH foaming with water and CO_2_ as co-foaming agents, and the results showed that the addition of appropriate water facilitated the polymer chains to become more flexible and improved the foaming performance. Salerno et al. used a mixture of CO_2_ and ethyl lactate to foam PCL and polylactic acid (PLA) and showed that the addition of ethyl lactate reduced the operating temperature and improved the morphological homogeneity of the foam [19]. Therefore, the use of co-foamers can improve the experimental operating conditions or improve the foam properties to some extent. The use of co-foamers has great potential in the manufacture of high-performance polymer foams [20].

## 3. Application Status

**Figure 4:**
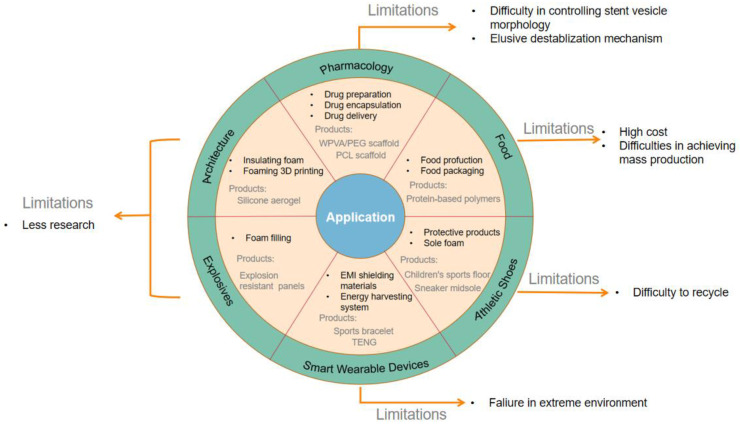
Application of supercritical foaming technology in various fields.

Supercritical foaming technology applies to many fields and applications in pharmacology, food, sports shoes, smart wearables, explosives, and construction are detailed below. Figure 4 shows the application of supercritical foaming technology in various fields.

### 3.1. Pharmacology

At present, the research of supercritical foaming technology in the medical neighborhood is a major hot spot. At this stage, the research mainly focuses on three aspects: drug preparation, drug delivery, and drug encapsulation. Among them, drug preparation is the main research direction. The specific applications of supercritical foaming technology in the medical field are as follows. (a) Foams containing uniformly dispersed active pharmaceutical ingredients can be used as a medium to gradually deliver the drug to the target organ or tissue [1]. (b) A drug such as a PCL scaffold prepared by supercritical fluid foaming combined with the leaching process to overcome the pore size adjustment problem of the supercritical foaming technique. This scaffold can facilitate bone repair by promoting osseointegration and avoiding post-graft complications [14]. (c) Preparation of plasticized poly (vinyl alcohol)/poly (ethylene glycol) (WPVA/PEG) scaffolds with a unique bimodal cell structure, which facilitates cell attachment and proliferation [21]. (d) Production of medical patches for skin injuries to ensure a favorable and sterile environment for the wound healing process [22]. (e) Using supercritical foaming technology can encapsulate the active ingredient in a drug, such as a microporous PLGA scaffold [23]. Current research on supercritical fluid foaming and drug preparation is focused on obtaining polymer foams with higher expansion rates, such as PLA foams by supercritical fluid foaming in combination with other techniques [24], as well as improving mechanical properties, such as the compressive strength of scaffolds, by this technology [25]. The application of supercritical foaming technology in the medical field is made possible by the use of non-toxic and non-hazardous foaming agents.

Conventional CBAs have many drawbacks: (a) they produce foams with high residues; (b) their main exothermic reactions make the foaming process conditions and the final pore structure difficult to control; (c) they are harmful to the user, causing skin, eye, and respiratory irritation as well as allergic reactions [11]. These drawbacks limit the application of CBAs in the medical field. However, PBAs are different. For example, SCFs can avoid these drawbacks. The commonly used ScCO_2_ and ScN_2_ are non-toxic, non-flammable, and chemically inert, and the residues are easy to remove [11]. This suggests that supercritical foaming technology plays an important role in the medical field.

However, controlling the size, shape and spatial distribution of scaffold pores using foaming techniques remains a key limiting step in the production of scaffolds [26]. In addition, further understanding of the destabilization mechanisms of polymer foams in contact with porous surfaces, such as skin or skin-like membranes, remains elusive [27].

### 3.2. Food

In the food sector, supercritical fluids are used in food production and food packaging. In food production, SCFs are mainly used in supercritical extraction to optimize food composition, improve food flavor, remove harmful substances, etc. [28,29]. Compared with supercritical extraction technology, supercritical foaming technology is less used in food production, but it is still in use. In the current study, the food products that were produced using supercritical foaming technology were mainly protein-based polymers. For example, the foaming properties of egg white protein (EWP) are easily influenced by protein concentration, protein composition, pH value, temperature, ionic strength, etc. However, if the foaming properties of EWP are improved by heat treatment, ultrasonic, chemical treatment, enzyme modification, etc., it may lead to chemical residues or affect the taste of food. Supercritical fluids, such as supercritical carbon dioxide, as green and non-toxic gas with good foaming properties can solve the above problems. The high-pressure and supercritical carbon dioxide synergistic treatment can provide a new way to improve the foaming capacity of EWP [30].

In food packaging, thermoplastic polymer foam is widely used due to its thermal insulation, high strength, and low density. This material provides lightweight insulation properties for packaging. The most common polymer foam used in food packaging is expanded polystyrene (EPS). However, EPS has environmental problems because it is non-degradable and difficult to recycle. Polyolefin foam, represented by polypropylene (PP) foam, is also widely used in food packaging and, although it poses some environmental hazards, is considered a promising polymer foam due to its low cost and recyclability [31]. In addition, another concern in this area is the consumer demand for foods with a longer shelf life. Carvacrol (CRV) is a phenolic essential oil with a wide range of biological and pharmacological properties, such as antibacterial, antioxidant, and anti-inflammatory activities. Foam samples containing higher CRV content exhibited antibacterial activity against spoilage and pathogenic bacteria [32]. However, the common foaming method used to produce the above-mentioned polymer foam for food packaging is chemical foaming, and supercritical foaming technology is rarely used. The main reason for this is the cost limitation, and the technology has a long way to go to achieve the mass production of food packaging. The advantages offered by supercritical foaming technology have not yet been effectively exploited in the food field.

### 3.3. Athletic Shoes

Supercritical foam technology is more widely known in the field of sneakers, as reflected in the foam material used in sneakers and the technology. With the maturity of supercritical foam technology, the shoe elastomer that can be applied expanded from TPU, PEBA to EVA, TPE, etc. Currently EVA (ethylene vinyl acetate copolymer) material is one of the important foaming materials in the field of sneaker manufacturing. This is attributed to its advantages of being lightweight, soft, anti-aging, non-toxic, good elasticity, tear resistance, and low-price of co-blended foam sneakers [33]. EVA has been used in a variety of applications, including shoe soles, sports equipment, and insulation [34]. The application of supercritical foaming technology to produce EVA materials requires attention to the issue of cross-linking. EVA molecular chains are linear and require a cross-linked structure to lock in the gas. At this stage, there is research on EVA foam materials, mainly focusing on the use of supercritical foaming technology to improve all aspects of foam performance by cross-linking or blending with other compounds. For example, recyclable EVA composite foam was successfully prepared by ScCO_2_ molding foaming of ionic cross-linked EVA composites, which also have smaller bubble sizes, larger bubble density, and resistance to permanent deformation in compression [33]. EVA was blended with PU to improve the wear characteristics of EVA foam [5]. Supercritical foam technology helps drive sneakers in a more comfortable and sustainable direction.

However, Hu Shengfei et al. showed that most of the EVA foam, rubber foam, and PU foam used in cushioning materials for shoe soles today have harmful residues to humans, poor elasticity and permanent deformation, and other problems. Moreover, it is difficult to recycle and easy to cause environmental pollution. They also showed that thermoplastic polyurethane bead foam (ETPU) has the advantages of being colorless, tasteless, environmentally friendly, heat-resistant, and wear-resistant, wear-resistant, non-slip and shock-absorbing, antibacterial and breathable, and highly resilient among other advantages. This material can be used in the production of sports shoes’ midsole and insole, but also in the production of plastic runways, children’s sports flooring, protective products, and so on. However, there are studies on this aspect of foam recycling. The recycling of waste foam usually includes mechanical recovery, chemical material recovery, and energy recovery. Among them, mechanical recycling is relatively simple. Coswald S. Sipaut et al. showed that the blending of virgin polymers (EVA) and used polymers (e.g., PU waste foam) can be considered a promising approach for the ecological and economic exploitation of polymer waste [34].

### 3.4. Smart Wearable Devices

With the advent of 5G and the internet era, wearable electronics with integrated mechanical flexibility and electronic functionality are attracting strong research interest in academic and industrial fields worldwide [35]. Supercritical foaming technology has also been widely used in this field. With the rapid popularity of wearable and second-generation flexible electronics, there is an urgent need for flexible and efficient electromagnetic interference (EMI) shielding materials to meet the requirements of electromagnetic (EM) protection [36]. Jianming Yang et al. investigated the preparation of lightweight flexible silicone rubber/silver plated hollow glass microspheres (HGMs) EMI shielding composite foams with gradient structure by supercritical CO_2_ foaming technology. Such composite foams have great potential applications in the field of EMI protection for wearable, smart, and sophisticated electronic devices [36]. In addition to electromagnetic interference problems, electronic devices have the problem of heat. EMI and high heat can reduce device performance and cause harmful effects on human health. Related studies have shown that a flexible composite material can be developed by introducing a three-dimensional (3D) liquid metal (LM) network into elastomeric foams. The material exhibited significantly improved electrical/thermal conductivity and EMI shielding efficiency (SE) under compressive conditions [37]. In addition, ultra-long time use is one of the keys to future application scenarios of wearable electronics, and the emergence of piezoelectric materials provides new ideas for self-powering electronic devices by harvesting mechanical energy from the surrounding environment. Here, polyvinylidene fluoride (PVDF) foam with a layered structural porous structure prepared by PVDF and NaCl can monitor human motion at different magnitudes of strain and frequency while collecting energy in a multidimensional stress field [35]. As we can see, supercritical foaming technology is essential to improve the performance of wearable devices.

Moreover, supercritical foaming technology can be used to prepare a triboelectric nanogenerator (TENG), a promising energy harvesting system. TENG is a rubber/carbon nanofiber composite obtained by a supercritical CO_2_ foaming process, which has the advantage of being environmentally friendly and suitable for mass production. TENG based on flexible silicone rubber can collect complex mechanical energy, such as stretching, twisting, bending, and compression. When affixed to the sole of a shoe, it can derive mechanical energy from human motion. Its output can also be considered a sensing signal for analyzing gait and sense motion. Extruded supercritical CO_2_ foam can prepare strip samples to collect energy on tires. Supercritical CO_2_ foam injection molding can design the mold according to the desired product, and the sole shape sample obtained by the injection molding method can collect energy from human movement. Therefore, the use of supercritical CO_2_ foaming may be a reasonable method to prepare fully shaped and adapted, flexible TENG with a porous structure [16].

However, although polymer foams exhibit excellent overall performance in everyday applications, these properties may fail under extreme environmental conditions, such as humidity changes, liquid flow, or entanglement. This can be optimized by encapsulation methods and should be further investigated [37].

### 3.5. Explosives

Explosives are essential in both the military and civilian industries. Supercritical CO_2_ foaming technology can also be applied to the preparation of infused explosives from spent explosives [38]. Mortars are the world’s major land warfare weapons and commonly generate energy from SP double-based propellants. However, a large number of discarded double-based propellants, such as SP double-based propellants, have been retired from service in various major military countries. Therefore, there is an urgent need to investigate formulations and technologies for the preparation of explosives from these dual-based propellants. The supercritical CO_2_ foaming process can facilitate the disposal technology of waste double-based propellants and can optimize the blast performance of the infused explosives by increasing the supercritical CO_2_ pressure [39]. In addition, polymer foams can be used as fillers for blast-resistant panels. For example, polyvinyl chloride (PVC) foam can be used as a filler in stainless steel corrugated core sandwich panels to improve their blast resistance. Foam fillers can greatly improve strength and energy absorption [40]. However, less research has been conducted on polymer foams for the explosives field.

### 3.6. Architecture

The intensive introduction of favorable policies for green building construction materials has expanded the application areas for natural insulation and energy-saving materials with heat insulation, thermal insulation, and sound insulation. At present, the green foam building materials that can be obtained by foaming technology mainly include foam glass, foam concrete, calcium silicate foam, expanded perlite, and expanded clay, etc. [41]. Compared with traditional foaming technology, the comprehensive performance of the foam obtained by supercritical foaming technology is excellent. It has excellent heat insulation, shock absorption, waterproofing, and sound insulation functions. With the globalization of green energy-saving buildings, improving the insulation performance of building facades has become an important breakthrough in reducing building energy consumption. In addition, the building envelope should be improved insulation performance as the starting point of energy saving. Zihao Song et al. developed a new material with excellent thermal and moisture barrier properties, namely silica aerogel. Silica aerogel is a high-performance insulation material and is a good solution for high energy consumption in building envelopes [42]. In conventional construction, foams are mainly used as insulation materials. Insulation materials help to improve the energy efficiency of buildings and reduce CO_2_ emissions. Rigid polyurethane foams are known to have excellent mechanical strength, chemical stability, high heat resistance, and relatively low environmental impact. In parallel, the construction industry has witnessed the development of reinforced and unreinforced material foam composites to improve the thermo-mechanical properties of foams [43].

Moreover, as far as the technology itself is concerned, foam 3D printing technology has created a boom in the construction industry. The geometric complexity offered by 3D printing technology has made possible the design of free-form architectural elements, which paves the way for a higher degree of customization. Moreover, 3D printing technology is an automated method that can increase labor and efficiency and improve worker safety [41]. However, the technology has not yet been studied enough in the construction industry and needs to be further improved in the future. The supercritical foaming technology is also not much used in the production of these green building materials, and if the advantages of supercritical foaming technology are incorporated, it is believed that the material performance will be optimized to a certain extent.

## 4. Assessment of Enabling Technology Development

**Figure 5:**
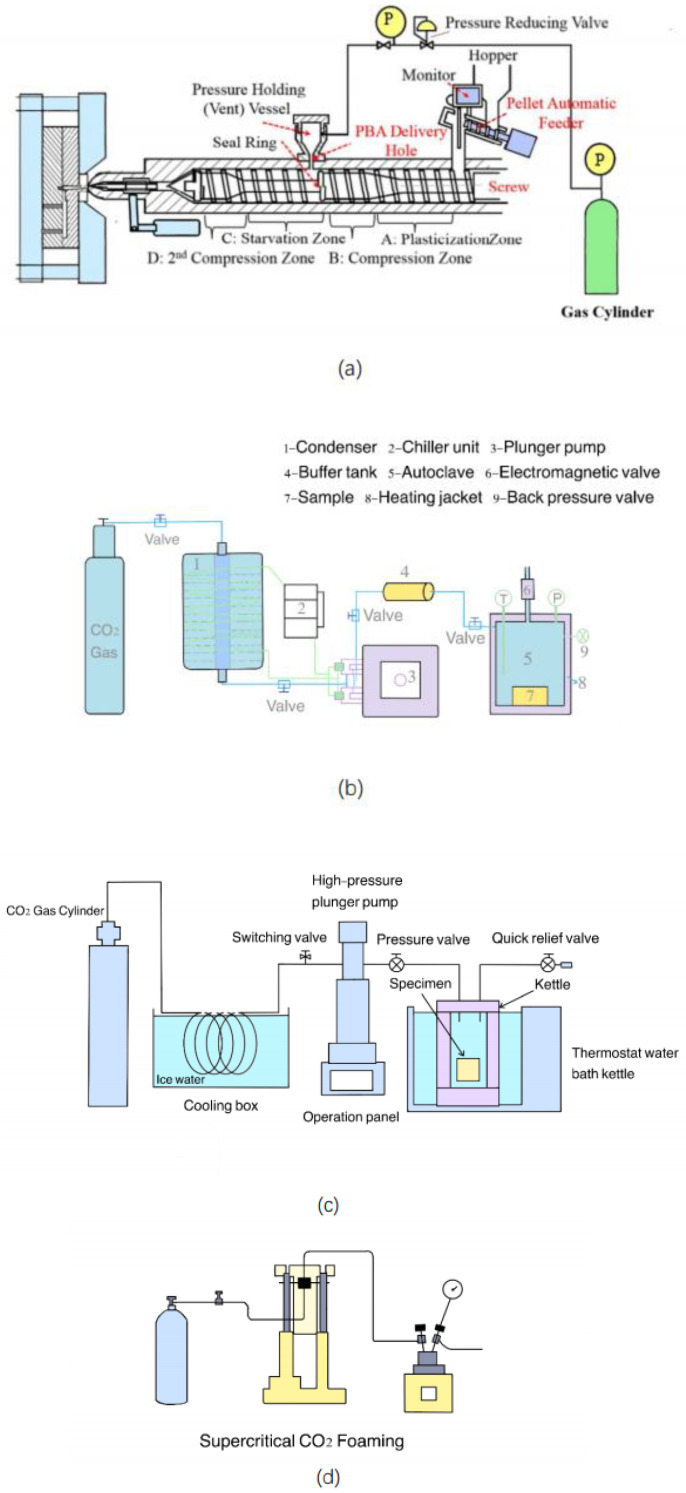
Related experimental flow chart: (**a**) RIC-FIM Technology. Adapted with permission from Ref. [15]. Copyright © 2017, American Chemical Society; (**b**) Supercritical foam combined with radiation crosslinking. Adapted with permission from Ref. [44]. Copyright © 2017, Wiley Periodicals, Inc.; (**c**) Supercritical foaming combined with particle leaching. Adapted with permission from Ref. [49]. Copyright © 2018, Springer Science Business Media, LLC, part of Springer Nature; (**d**) Supercritical foaming combined with 3D printing. Adapted with permission from Ref. [51]. Copyright © 2021, American Chemical Society.

Supercritical foaming technology has played a great role in the preparation of polymer foams. Improvements in the application of this technology are still being made. The following are the current technical supports that favor the development of supercritical foaming technology. Figure 5 shows a diagram of the apparatus used for each improved technique. The favorable technologies and developments are listed in Table 1.

### 4.1. RIC-FIM Technology

Regarding the improvement of the technology itself, Long Wang et al. developed a resilient and innovative cellular foam injection molding (RIC-FIM) technology. In the preparation of microcellular foams, it is not necessary to pressurize a PBA such as CO_2_ to a supercritical state. The team later built on this and further simplified the device to reduce costs while still maintaining foaming properties and producing fine microporous structures. This simplified FIM machine provides a viable and promising method for manufacturing lightweight injection molded products that are cost-effective and easy to handle [15]. If this technology can be widely used in the future, it will undoubtedly reduce the cost to a great extent.

### 4.2. Combined with Cross-Linking Technology

It has been shown that an appropriate ionic cross-linking degree is beneficial to increase the volume expansion and bubble density of the resulting foam and reduce the average bubble diameter and compression permanent deformation of the foam [33]. Currently, supercritical foaming technology has been used in combination with cross-linking technology to prepare polymer foams with high density and good resilience. At present, the more studied crosslinking method is radiation cross-linking. The so-called radiation crosslinking refers to the technical means of using various kinds of radiation to initiate crosslinking reactions between the long chains of polymer polymers. For example, closed-cell polypropylene (PP) foams can be prepared by supercritical carbon dioxide foaming with the help of γ-ray radiation cross-linking. Polymer foams with closed-cell structures have high mechanical properties and excellent thermal, acoustic, and water resistance properties [44]. In addition, the preparation of fluoroelastomer foams has been a promising task due to their susceptibility to shrinkage. A promising approach to overcome this challenge is the implementation of synergistic techniques, i.e., a combination of irradiation pre-crosslinking, supercritical fluid foaming, and supercritical N_2_ oven secondary cross-linking [45]. The combination of irradiation crosslinking techniques contributes to foaming properties and the formation of well-defined cell structures [46].

### 4.3. Combined with Particle Leaching Technology

Combined with particle leaching technology manufacturing can overcome the pore size adjustment problem of supercritical foaming technology, manufacturing foams with precise pore structure and high porosity, which has a broad application prospect in the field of tissue engineering. For example, a bimodal porous scaffold was designed for bone tissue engineering (bTE) by combining supercritical CO_2_ foaming technology and pore-former leaching technology. A drugged PCL scaffold was prepared by supercritical foaming technology combined with a leaching process to overcome the pore size adjustment problem of supercritical foaming technology. A bimodal pore structure (including macropores and micropores) of poly (lactic acid)-hydroxyacetic acid copolymer (PLGA) scaffolds was prepared by combining supercritical CO_2_ foaming technology with particle leaching technology, etc. In combination with the leaching technique, finer pore size and more uniform distribution of foams can be obtained, thus ensuring the higher porosity and open pore ratio of the prepared scaffolds [14,47,48,49].

### 4.4. Add Filler

The addition of fillers is one of the methods that have great potential to improve the properties of polymer foams. This strategy not only offers foaming advantages, but also improves the properties of the final composite. In general, fillers change the foam pore structure and mechanical properties of foams, but each type of filler produces specific effects depending on its properties. Therefore, the role of fillers in the foaming process and foam morphology still needs further research and understanding [11].

### 4.5. Combined with 3D Printing Technology

Supercritical foaming technology combined with 3D printing technology allows the production of polymer foam products with complex structures. This feature makes it possible for foam products to meet the needs of specific forms in a wider range of fields (e.g., construction, medical, etc.). At the architectural level, the geometric complexity provided by 3D printing technology makes possible the design of free-form architectural elements that are difficult to produce with other technologies [41]. At the medical level, the combination of supercritical foaming technology and 3D printing allows the production of bone scaffolds that are more responsive to patient needs [50]. At the level of piezoelectric devices, piezoelectric energy storage with a biomimetic wood structure is prepared by 3D printing and supercritical CO_2_ foaming [51]. However, foam printing technology has not yet matured and is expected to become a rapidly developing research area and industrial trend as a promising alternative to the technology of foam injection molding [52]. When this technology matures, the customization of foam products will be more easily achieved.

**Table 1:** List of favorable technologies and developments.

Technology	Development	Means of Implementation	Ref
RIC-FIM	Simplify installations and reduce costs	The transmission of PBA does not require high-pressure pumps or injection valves	[15]
Cross-linking	Increase the density of bubble pores and enhance the foaminess	Mainly radiation crosslinking	[44]
Particle leaching	Overcoming foam pore size adjustment problems	Porogen leaching techniques.	[48]
Add filler	Change the bubble structure and mechanical properties of the foam	Add wood flour or other filler	[11]
3D printing	Production of foam products with complex structures	Selective laser sintering (SLS) printing and more	[51]

## 5. Remaining Challenges and Future Research

Supercritical foaming technology has now become one of the most important technologies in major fields. Despite recent technological advances and efforts, many challenges remain before the technology can be truly applied. The followings are the challenges and issues that need to be addressed in the future of supercritical foaming technology.

### 5.1. Technical Limitations

Although many methods have been researched to control the bubble hole morphology, size, and spatial structure to produce foams that meet the demand, these control methods are specific. This means that continuous research will be required in the future as the control methods vary depending on the production requirements. The existing control methods do not meet all production needs. As for the ScCO_2_ foaming technology, although it has more advantages compared to other chemical foaming methods, it also has limitations: special equipment is required, the foaming of semi-crystalline polymers is low, and the saturation time is usually long [53]. Therefore, improvements in supercritical foaming technology should be carried out continuously.

### 5.2. Limitation of Foaming Agent

At present, the foaming agents used in supercritical foaming technology are only ScCO_2_ and ScN_2_. CO_2_ and N_2_ are common gases and their sources vary widely. CO_2_ can be obtained by carbon capture technology and N_2_ can be obtained by fractional distillation of liquid air. Therefore, the cost of foaming agents for supercritical foaming technology is lower than that of chemical foaming agents. Especially CO_2_ is the main foaming agent, and there is a lot of research on supercritical CO_2_ foaming technology. Then, we have to think about whether other supercritical gases can also be used in supercritical foaming technology. For example, can rare gases, which are also inert in the supercritical state, be used for supercritical fluid foaming? Preliminary studies have been conducted on krypton and argon mixtures as foam insulation foaming agents, but the technology has not been widely adopted. This may be due to the significant leakage rate and ambient air replacement that the foam undergoes after fabrication [54]. There are very few studies on this aspect at this stage, and it remains to be confirmed whether other supercritical gases can be used for foaming. There is a strong link between the type of foaming agent and the properties of the foamed product. So, if we can broaden the types of foaming agents, we will get more products with different properties.

### 5.3. Technology Cost

Although the foaming agent cost of supercritical foaming technology is lower than that of chemical foaming technology, the equipment used in supercritical foaming technology such as Mucell equipment costs more than ordinary equipment. For example, the investment in Mucell equipment is 35% higher than that of ordinary equipment, which will limit the application of the technology to a certain extent (Source: China CNC machine tool network). Although the corresponding simplification technology has been developed for the equipment, it has not yet been put into production. Therefore, improved solutions that can both reduce costs and increase foaming success need to be developed and put into production. This will provide the basis for achieving the widespread use of supercritical foaming technology.

### 5.4. Recycling

The use of supercritical foaming technology reduces pollution in the production process and avoids the environmental and human hazards of the foaming agent itself. However, if waste products such as EVA polymers cannot be recycled in time, they will still cause damage to the environment. However, a complete recycling mechanism has not yet been established. It is therefore imperative that this issue is resolved. Otherwise, the green development of supercritical foaming technology cannot be achieved in a real sense.

## 6. Conclusions

Although the concept of supercritical foaming technology is not new, its practical application is still limited. However, with environmental issues becoming more prominent and green chemistry becoming an important means of addressing environmental energy issues today, the time seems right for the widespread application of supercritical foaming technology. Although there has been some improvement and development in supercritical foaming technology, there are still some challenges that need to be addressed to ensure that the technology can be put into practical production on a larger scale. This includes breaking through the limitations of the technology itself, broadening the range of foaming agents, reducing the cost of the technology, and establishing a complete recycling mechanism. Supercritical foaming technology has been used in athletic shoes, pharmacology, and the preparation of some small devices, but this is not enough to fully exploit the benefits of the technology. Supercritical foaming must break away from its current limitations and move towards a wider range of applications. The most promising areas are currently in the field of pharmacology, where the technology can be used to produce scaffolds with excellent properties for bone tissue engineering. However, other areas, such as food, smart wearables, explosives, and construction, are not yet ripe for research into supercritical foaming technology. Further research is needed to truly implement supercritical foaming technology into actual production.

## Data Availability

Data is contained within the article.
